# Calculator for inadequate micronutrient intake for Ethiopia (CIMI‐Ethiopia): Validation of the software for lactating mothers and their children under 2 years

**DOI:** 10.1002/fsn3.2922

**Published:** 2022-06-03

**Authors:** Beruk Berhanu Desalegn, Christine Lambert, Ute Gola, Simon Riedel, Tegene Negese, Hans Konrad Biesalski

**Affiliations:** ^1^ 128167 School of Nutrition, Food Science and Technology Hawassa University Hawassa Ethiopia; ^2^ Institute of Nutritional Sciences University of Hohenheim Stuttgart Germany; ^3^ Day‐med‐concept GmbH Berlin Germany

**Keywords:** children, CIMI‐Ethiopia, Ethiopia, lactating mothers, nutrient inadequacy, NutriSurvey

## Abstract

Early identification of inadequate intake of nutrients from a person's diet is usually crucial to prevent the development of micronutrient malnutrition. However, there is no single dietary assessment tool for Ethiopia that can assess the nutrient intake of a person from the type of food she or he consumed with a given amount. Therefore, the Calculator for Inadequate Micronutrient Intake (CIMI) application was adapted in consideration of food and nutrition contexts in Ethiopia and validated for its suitability to compute nutrient intake and identify nutrient intake inadequacy. For this, a 24‐h recall quantitative dietary data of children aged 12–23 months (*n* = 781) and lactating mothers (*n* = 1086) were collected between February 15 and 30, 2017, from rural Genta Afeshum district, Tigray region, Ethiopia. An individual nutrient intake was estimated by calculating using CIMI‐Ethiopia and also by NutriSurvey (NS) software for comparison. The average (mean and median) intake of energy and most nutrients and the prevalence of inadequacy calculated by the two software for the children aged 12–23 months and lactating mothers were comparable, except that of the vitamin A. The correlation coefficients for the intake results calculated by CIMI‐Ethiopia and NS were between 0.85 and 0.97 for the children and between 0.5 and 0.96 for the lactating mothers' group. Most of the mean intake differences calculated by the two methods were within the acceptable limits, except for the vitamins A, D, and B12 in the Bland–Altman plots. CIMI‐Ethiopia is very sensitive to identifying energy, protein, and selected micronutrients inadequacy included in this study, both for the lactating mothers (84.1%–100%) and 12–23‐month‐old children (77.6%–100%) group. Our results showed that CIMI‐Ethiopia estimates the energy and nutrient intake, and can be also used as a screening tool to identify energy, protein, and selected micronutrients inadequacy from an individual woman's and child's diet in rural Tigray, Ethiopia.

## Graphical abstract

Calculator for inadequacy.

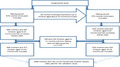

## 1 INTRODUCTION

Micronutrients are important nutrients that are only required in minimal amounts to support the growth, development, organ function, immunity, metabolism, and maintenance of the human body (Bhandari & Banjara, 2015). However, factors such as inadequate intake from diet, poor bioavailability, not meeting the additional need due to disease‐related losses or increased need, and extra requirement for physiologically needed groups including pregnancy and lactation period, lead to an invisible form of malnutrition called micronutrient deficiency (MND) or ‘hidden hunger’ (Biesalski Hans & Jana, 2018). Because the manifestations of this deficiency problem are less visible shortly, MND has been identified when the condition reaches a severe level and also after the health of the individual is affected seriously. MND is among the important public health problem globally and affects >2 billion people, primarily females, and children (Biesalski Hans & Jana, 2018). It was also identified as the principal cause of a significant number of maternal and childhood morbidity and mortality (Black et al., 2008). Therefore, its impact on the social, economic, and health status of society and countries is immense. To prevent the devastating effect of MND on human life, early identification of inadequate intake of nutrients from a person's diet is usually crucial. However, there has been less attention given to the early diagnosis of micronutrient deficiency. Besides this, groups who can be at risk for hidden hunger often are overlooked, due to a lack of easy‐to‐handle assessment tools. Thus, intervention programs are implemented lately (Jati et al., 2014). This is more serious in developing countries, due to the population‐level dietary intake information is obtained from national‐level household socioeconomic surveys. But, these socio‐economic surveys are not primarily designed to collect dietary nutrient intake data, and they are conducted at a long time interval, time taking, and also costly. However, individual dietary intake surveys are more important to collect detailed information, but most of the dietary nutrient intake assessment techniques are time‐consuming, need well‐trained data collectors, and require a well‐experienced data encoder to accurately estimate the portion sizes eaten at the individual level (Jati et al., 2014).

Furthermore, they require individuals with experience in analyzing the dietary intake and identifying the level of inadequate micronutrient intake for an individual or at the population level from their diet using dietary assessment software and identifying inadequacy. Alternatively, scholars in Ethiopia and other developing countries have been using different types of diet diversity scores (i.e., infant and young children minimum diet diversity score women's diet diversity score and minimum dietary diversity for women of reproductive age) to be computed from the qualitative dietary information as a proxy indicator of micronutrient adequacy for individuals (Azene et al., 2021; Bitew et al., 2021). Thus, developing and validating friendly, fast, and offline usable applications that can measure the nutrient intake and detect the gap between the actual nutrient intake and the recommended intake is an important step. This helps to easily measure the burden of micronutrient intake inadequacy and evaluate to monitor the effectiveness of interventions that are targeted to improve micronutrient intake.

The Calculator for Inadequate Micronutrient Intake (CIMI) is an informative web‐based application software, which was first developed for Indonesian people to calculate the iron, zinc, and vitamin A intake in comparison to recommended nutrient intake (RNI) (Jati et al., 2014). Then, it was also adapted to Ghanaian and Tanzanian contexts (Lambert et al., 2018; Wald et al., 2019). CIMI can be used to calculate energy, protein, and micronutrient intake from the diet consumed by an individual using food groups constructed for a specific CIMI version. It also identifies nutrient inadequacy of an individual using Food and Agriculture Organization/World Health Organization (FAO/WHO) RNI for different age, sex, and physiological stages immediately after individual dietary intake data entry. Exceptionally, CIMI also calculates the bioavailability of iron and zinc in the diet of an individual to identify the inadequacy of the specific micronutrients. For this, an algorithm was constructed to classify iron and zinc bioavailability. In this case, the various scenario of bioavailability was taken into account, which included 5%, 10%, 12%, and 15% for iron bioavailability and high, medium, and low for zinc bioavailability, which is based on the RNI from the FAO/WHO (Jati et al., 2014). Therefore, CIMI helps to provide dietary counseling on dietary behavior immediately based on the intake gaps observed in consideration of food items available in the locality (Jati et al., 2014). Once the individuals' dietary intake data are collected using the CIMI application in the field, and whenever the tablet obtains a Wi‐Fi network, the whole data can be synced immediately to a server. Then, these data will be available and can be used for both individual‐ and population‐level dietary consumption interpretation using different dietary reference recommendations. Besides these, CIMI is designed to calculate nutrient intake from an individual diet using a 24‐h dietary assessment method (Bosha, Desalegn, et al., 2019; Bosha, Lambert, et al., 2019; Jati et al., 2014; Lambert et al., 2018; Wald et al., 2019).

CIMI‐Ethiopia has been adapted to the Ethiopian context considering the dietary pattern of Ethiopian people by including commonly consumed 93 food items, which are aggregated into 31 food groups. It is released as a mobile application for use with a tablet, to collect multiple 24‐h quantitative dietary recall information. CIMI‐Ethiopia also supports other dietary data collection methods like weighted food records to calculate nutrient intake from individual dietary data. It also included pictures for each food item of the CIMI‐Ethiopia food database to support identifying and recalling some types of food items, both for the interviewer and the interviewee. To increase the precision for estimation of the amount consumed by the individuals, local units which are known across different areas of the country were identified. Once this was completed, 15 sliding intervals representing a certain amount by considering the minimum and maximum amount estimated to be eaten by the children and adults for reducing recall bias were identified and included in the CIMI‐Ethiopia. The intervals with the corresponding amount values will help to ease the estimation of the amount of food consumed from each food group constructed for CIMI‐Ethiopia.

Cereals and root‐ and tuber‐based foods are staples for Ethiopians; thus, considering this for its full usage of CIMI‐Ethiopia is important, but it needs to evaluate the validity of the results generated by the software in the two contexts. Accordingly, CIMI‐Ethiopia was validated using dietary intake data collected from women of reproductive age, in southern Ethiopia, where root‐ and tuber‐based diets are staples in the community (Bosha, Desalegn, et al., 2019; Bosha, Lambert, et al., 2019). The study revealed that CIMI‐Ethiopia precisely calculates nutrient intake for women of reproductive age in southern Ethiopia. Yet, it is not known, where CIMI‐Ethiopia produces valid results in terms of calculating the nutrient intake and also identifying the inadequate intake of specific nutrients from individual dietary information. Moreover, the validation study in southern Ethiopia was based on data collected during different agricultural seasons, unlike in northern Ethiopia, in which the dietary pattern is profoundly affected by religious fasting (Bosha, Desalegn, et al., 2019; Bosha, Lambert, et al., 2019; Desalegn et al., 2018; Desalegn et al., 2019). Besides this, the validity of CIMI‐Ethiopia for its ability to calculate the precise results based on children's data is not assessed.

Therefore, this study was initiated to assess the validity of the results generated by CIMI‐Ethiopia based on the dietary intake data of lactating mothers and their 12–23‐month‐old children collected from the Tigray region, northern Ethiopia, compared to established nutrition assessment software, NutriSurvey.

## 2 MATERIALS AND METHODS

### 2.1 Study setting, design, participants, and periods

The study was conducted in rural areas of the Genta Afeshum district of Ethiopia. It is one of the hot spot areas of food insecurity in the region. In the district, a total of 99,112 people were estimated to be living, including both rural and urban residents, of which 99% were followers of the Ethiopian Orthodox religion. The district covers an estimated area of 1636 km^2^ which exists with an altitude between 2045 and 3314 meters above sea level (Federal Republic of Ethiopia, 2015; Ludwig & Bau, 2007; Tigray Region Agriculture & Rural Development Bureau, 2016; WFP, 2009).

**FIGURE 1:**
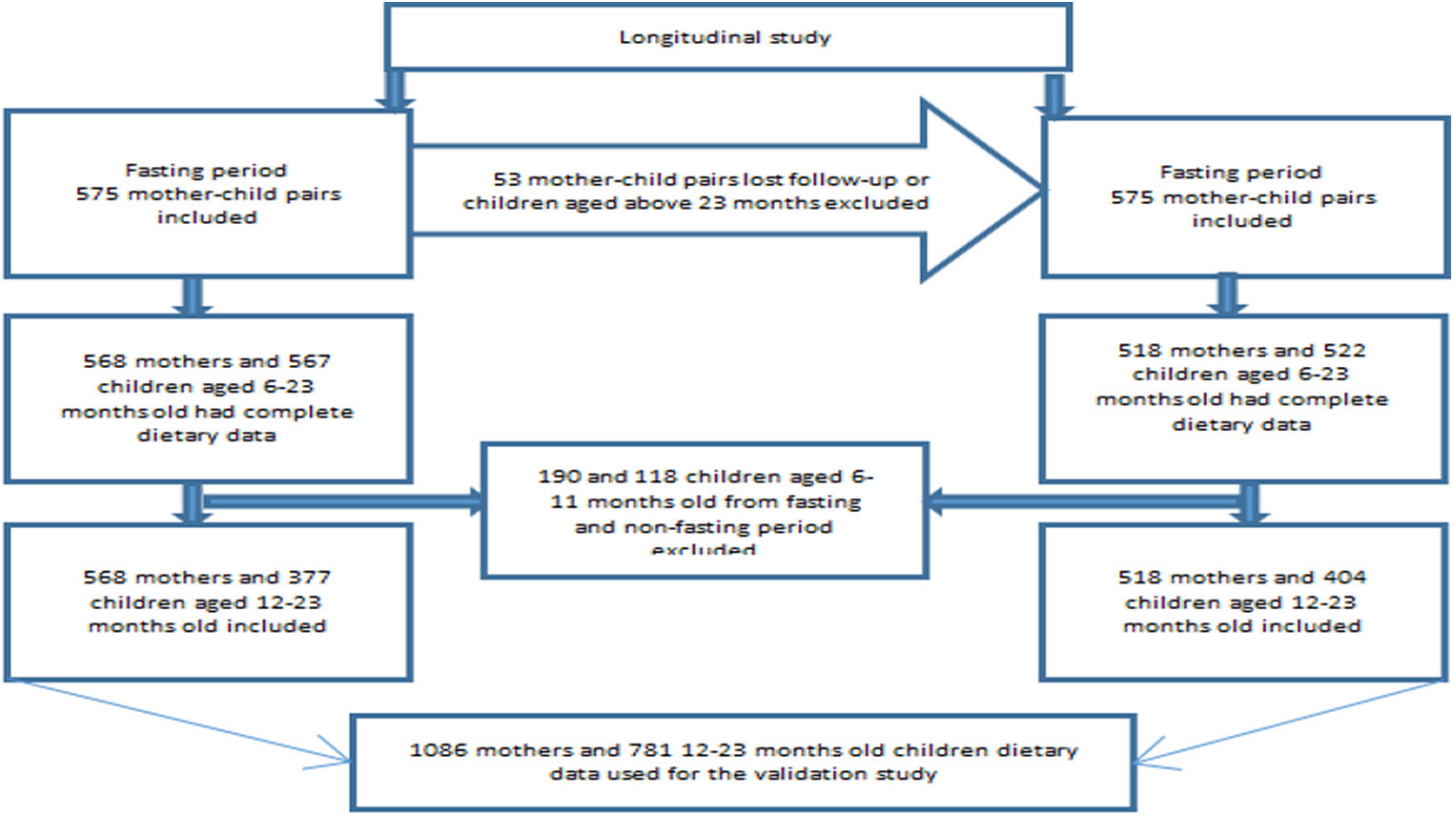
Schematic representation showing the sampling procedure for the validation study

This study was a component of a longitudinal study that was aimed to assess the impact of fasting on dietary patterns and nutritional status of pairs of lactating mothers and 6–23‐month‐old children (*n* = 575 for lent fasting period and *n* = 522 for nonfasting period) (Desalegn et al., 2019). Therefore, the sample size was calculated for this specific validation study, with a two population proportion formula using OpenEpi Version 3.01, with an assumption of a 10% prevalence difference calculated by the two methods for inadequate intake of iron among 12–23‐month‐old children, a confidence level of 95% and power (80%). Thus, the calculated sample size was 385. However, the sample size calculated for other specific objectives was high; thus, a large sample size was considered for this study. Moreover, for the present study, the children aged 6–11 months during either of the two surveys (fasting and nonfasting periods) were excluded. But those children who were below 12 months of age in the lent fasting but aged 12 or more months old in the nonfasting period were included in the study. Thus, the final number of data cases for 12–23‐month‐old children included in this study was 781(*n* = 377 for lent fasting and 404 for nonfasting) from children who participated in the lent fasting and nonfasting assessments, respectively. Likewise, among the data cases of lactating mothers who participated in the longitudinal study, those who had incomplete data were also excluded. Therefore, a total of 1086 data cases (*n* = 568 for lent fasting and 518 for nonfasting) of lactating mothers were included in this study (Figure 1). The dietary data used for this study were collected during the Ethiopian Orthodox lent fasting (February 15–April 15, 2017) and nonfasting (May 1–30, 2017) periods.

### 2.2 Data collection

Food items, recipes, cooking, and serving materials, as well as how the commonly consumed foods were prepared for children and lactating mothers, were identified before conducting the actual survey. Pictures of common dishes and serving utensils were used to help the mothers to estimate the amount of food their children consumed. Weights of food items were measured using food weighing scales (Model CS 2000, Ohaus Corporation). An English version dietary assessment questionnaire was adapted and translated to Amharic, and later to the local language Tigrigna (Gibson & Fergusen, 1999). Two data collection training were given to the data collectors on the topics such as basic dietary information, 24 h quantitative dietary recall data collection method, and the procedures to be followed using an interactive approach coupled with practical data collection. Then, the necessary amendments were made and checked for their appropriateness by the data collectors and approved by the principal investigator after the Tigrigna version questionnaire was pretested at the community level. An interactive quantitative 24 h dietary recall was implemented using a multiple pass technique which was adapted for use in developing countries such as Ethiopia, to collect quantitative dietary data of the mothers and children for the last 24‐h period (Gibson & Fergusen, 1999).

### 2.3 Development of calculator for inadequate micronutrient intake for Ethiopia (CIMI‐Ethiopia)

Food items commonly consumed by Ethiopians were identified from the socioeconomic survey dataset based on the daily per capita consumption in kilogram to structure food groups for the CIMI‐Ethiopia program (CSA, 2016). In addition, literatures about food consumption pattern in Ethiopia were reviewed to support the food grouping work. Accordingly, 93 commonly consumed food items were identified, and the energy and macro‐ and micronutrient concentrations for each of the identified food items were obtained from the Ethiopian food composition tables III and IV (EHNRI, 1998; ENI, 1981). Concentrations for nutrients missing in the Ethiopian food composition tables were taken from the US Department of Agriculture USDA (2018) database by adjusting with the moisture contents of the food items, which was calculated as presented in Figure S1 (Appendix S1). Then 31 food groups were created from the commonly consumed food items (Table 1). The amount of food consumed per capita per day, the nutrient composition of the food items, and standard food grouping (e.g., green leaves, see Table 1) were considered in the process to form food groups for the CIMI‐Ethiopia program (Ayana et al., 2018; CSA, 2017; Demissie et al., 2010; Tessema et al., 2019; Tezera et al., 2018). Shape and volume of food items, sizes or volumes of local utensils or foods commonly used in local markets, and varietal differences in the few food items related to consumption pattern and specific micronutrient content were also considered for food grouping. For example, chard, lettuce, and Ethiopian kale were grouped under ‘green leaves’ based on their micronutrient contents (Fe, Zn, and pro‐vitamin A), shape, and because they are vegetables. Likewise, sweet potato, potato, taro, cassava, yam, *amicho* (boiled decorticated root part of false banana), and *anchote* (*Coccinia abysinica*) were aggregated based on their shape and standard food group (roots and tubers) into ‘potato and related foods’ group. Maize was grouped into two groups as white and yellow maize because this grain is highly consumed by the majority of people in Ethiopia and there is a high variation in the pro‐vitamin A content between them. Similarly, teff was also grouped into two as white and red, considering the consumption of the grain is high as that of the maize and the high content of Fe in the red than the white teff (Abebe et al., 2007). The minimum and maximum total amount of food items to be eaten by young children and adults from each of the food groups per capita per day were identified after food grouping work was completed. Then, sliders with 15 intervals were established for each of the constructed food groups representing different amounts of corresponding values for the local units to standardize them. Therefore, an individual respondent will be supported to estimate the total amount consumed from each food group based on the standardized local units established for each food group in CIMI‐Ethiopia.

**TABLE 1:** Description of the food groups in CIMI‐Ethiopia

Food group	Foods contributed to the calculation of the average nutrient content of the food group
White maize	White maize
Yellow maize	Yellow maize
Wheat	Wheat
Sorghum and millet	Sorghum and finger millet
Red teff	Red teff
White teff	White teff
Barley and related	Barley, rice, and oats
Beans	Haricot bean, broad bean, chickpea, pea, lentil, kidney bean, soybean, and vetch
Oilseeds	Linseed, niger seed, peanut, sesame seed, sunflower seed, safflower seed, amaranth seed, and white fenugreek
Kocho and bulla	*Bulla* and *Kocho*
Potato and related	Irish potato, sweet potato, yam, cassava, taro, anchote and *Amicho*
Meat	Goat meat, sheep meat, beef meat, and chicken meat
Fish	Fish
Egg	Chicken egg
Milk	Milk, cow
Cheese	Cheese, cow
Butter and oils	Butter, vegetable oil, and palm oil
Sugar and honey	Sugar and honey
Sugarcane	Sugarcane
Moringa	Moringa
Carrot and related	Pumpkin and carrot
Green leaves	Chard, lettuce, and Ethiopian kale
Green pepper and related	Green pepper and garlic
Pepper powder	Pepper powder
Other vegetables	Tomato, onion, cabbage, beets, and snap bean (*Fossolia*)
Mango and related	Mango, papaya, guava, and apricot
Banana	Banana
Orange and related	Orange, mandarin, avocado, pineapple, apple, strawberry, lemon, cactus, passion fruit, custard apple, watermelon, peach, and pomegranate
Coffee and tea	Tea and coffee
Coca cola and mirinda	Berez, coca cola, pepsi cola, fanta, mirinda, and sprite
Mild alcohol	Beer, *tella, tej*, and *borde*

The nutrient profiling for the 31 food groups was based on the share contribution of each food item for the specific CIMI‐Ethiopia food group, so that the food groups have their average profile for energy and other nutrients. For example, the sorghum and millet food group had a share of 57.4% and 42.6%, respectively, which means that the total energy and each nutrient for the specific group is the sum of the respective amount of energy or other specific nutrients obtained from sorghum and finger millet according to their share the food items contributed. Therefore, the total amount of energy and nutrient intake calculated by CIMI‐Ethiopia for a person is the sum of the respective energy and nutrient intakes obtained from all the food groups consumed. However, an algorithm for classifying the bioavailability of iron and zinc was adopted from CIMI developed for Indonesia (Jati et al., 2014). Thus, CIMI‐Ethiopia automatically calculates the absolute intake and percent of RNI fulfillment for energy and each of the other 11 nutrients (protein, calcium, magnesium, and vitamins A, B1, B3, B6, B12, B5, C, and D), and also iron and zinc considering the algorithm included in the study.

### 2.4 Data management and analyses

The 93 food items considered for CIMI‐Ethiopia with their nutrient composition were entered into NutriSurvey (NS) software version 2007 as database for analysis. Then, the estimated amounts of food items consumed by children and mothers within 24 h before the survey by the children and mothers involved in the study were entered corresponding to the food items database in NS and nutrient intake was calculated. However, for CIMI‐Ethiopia, the estimated amount of food items consumed by the children and mothers were sorted under the 31 food groups created, the estimated amounts consumed from each of the food items were summed up to give the total amount for the respective food group, and then the results from the summation were entered. Data were entered into CIMI‐Ethiopia using tables. The tablets with the data were synced with the server (University of Hohenheim, Germany) using a Wi‐Fi connection daily. Then, data were downloaded from the server and were imported into SPSS software version 20 (IBM Corporation). Data imported into SPSS were coded, checked for completeness, and cleaned.

Then, the results for energy and each nutrient intake results calculated by CIMI‐Ethiopia and NS application for every mother and child were compared against the respective RNI and Estimated Average Requirement (EAR) values to judge inadequate intake (Asayehu et al., 2017; Institute of Medicine (IOM), 2005, 2011; Moges et al., 2022). Specifically, iron and zinc vary because of the different bioavailability levels (i.e., for iron: 5%, 10%, 12%, and 15%; for zinc: low, moderate, and high) considered in CIMI‐Ethiopia; thus, the RNI cutoff point differs from individual to individual due to food matrices (Jati et al., 2014). Then, the prevalence of inadequate energy and nutrients intake was calculated based on the intake results computed by the NS and CIMI‐Ethiopia applications. Furthermore, the cumulative energy and nutrient intakes data were used to calculate the average energy and each nutrient intake for the two study groups separately (FAO/WHO, 2001; FAO/WHO/UNU, 2001a; Institute of Medicine (IOM), 2005, 2011).

To categorize the ability of CIMI‐Ethiopia for its ability to precisely calculate the nutrient intake compared to NS, the mean difference % level (i.e., between the two software) was calculated following a procedure adopted from Lambert et al. (2018). Accordingly, the average mean differences of each nutrient intake were calculated as an average of the mean difference results obtained by subtracting the results produced by NS results from CIMI‐Ethiopia of the participants included in the study, and the average mean difference % for each nutrient was calculated as the average mean difference divided by the average nutrient result produced by NS and then multiplying the dividend by 100 (Appendix S1, Equation a and b). Then, the percentage mean difference was used to categorize the accuracy of the results of energy and nutrient intake produced by CIMI‐Ethiopia as very high (±0 < 5%), good (±5%–15%), moderate (±15%–30%), and low (±>30%) (Bosha, Desalegn, et al., 2019; Bosha, Lambert, et al., 2019; Lambert et al., 2018).

The correlation between the results produced by the CIMI‐Ethiopia and NS was analyzed using Spearman correlation analysis. For this, energy and each nutrient intake calculated by CIMI‐Ethiopia and NS for the 12–23‐month‐old children and lactating mothers were entered into SPSS, and the correlation coefficient was calculated and the statistical significance was declared at *p* <.05.

The Bland–Altman method was used to examine the mean bias, limits of agreement, and distribution of bias over the range of energy and nutrient values calculated by CIMI‐Ethiopia and NS. For this, the average (X‐axis) of the mean values and their differences (Y‐axis) were calculated and used to draw Bland–Altman plots. It is recommended that 95% of the data points should lie within the limit of agreement (±1.96 *SD*) (Giavarin, 2015).

Moreover, the sensitivity % of CIMI‐Ethiopia to calculate energy, protein, and also for each micronutrient was calculated as the number of true inadequate (true positive) identified by CIMI‐Ethiopia divided by the summation of true inadequate (true positive) and false adequate (false negative) individuals, then the dividend multiplied by 100%. Similarly, the specificity % of CIMI‐Ethiopia to calculate energy, protein, and each micronutrient was calculated as the number of true adequate (true negative) identified by CIMI‐Ethiopia divided by the summation of true adequate (true negative) and false adequate (false positive) individuals, then the dividend multiplied by 100%. The positive predictive value of CIMI‐Ethiopia for each nutrient (including energy) considered in CIMI‐Ethiopia was obtained by dividing true positive over the sum of true positive and false positive, followed by multiplying the dividend by 100. For the negative predictive value, the true negative was divided by the sum of true negative and false negative, then the dividend was multiplied by 100 (Trevethan, 2017). The formulas used for calculating the sensitivity, specificity, and predictive value (positive and negative) of CIMI‐Ethiopia are presented as Equations C, D, E, and *F* (Appendix S1).

## 3 RESULTS

### 3.1 Sociodemographic characteristics

Of the total 12–23‐month‐old children included in the lent fasting (*n* = 377) and nonfasting (*n* = 404) period surveys, 166 (44.0%) and 182 (45.0%) children participated during fasting and nonfasting periods were females. The mean age for the children included during the fasting and nonfasting period was 16.8 ± 3.3 and 17.6 ± 3.5 months, respectively. Whereas, the average age of the lactating mothers was 29.8 ± 6.4 years, (data not shown).

### 3.2 Prevalence of inadequate intake of energy and nutrients calculated by CIMI‐Ethiopia and NS

The differences in the prevalence of inadequate intake of energy (0.2%) and most nutrients (*n* = 13) calculated by CIMI‐Ethiopia and NS for children regardless of the cutoff (RNI and EAR) used in this study were low (protein: 0.1%–0.7%; iron: 1.6%–2.5%; calcium: 0.2%; Mg: 1%–1.5%; vitamin B1: 0.1%–1.9%; Niacin: 0.1%–1.3%; vitamin B6: 2.7%–3.9%; vitamin B12: 0.5%–0.6%; Pantothenic acid: 4.7%; vitamin C: 1.4%–1.8%; and vitamin D: 0.4%–0.5%), except for the vitamin A (28.8%–45.2%). Likewise, the differences in the prevalence of inadequacy calculated from the results by CIMI‐Ethiopia and NS for the lactating mothers group were low for energy (4.2%) and majority of nutrients (protein: 0.4%–1.4%; iron: 0.1%–0.9%; zinc: 1.3%–6.7%; calcium: 0.4%–1.5%; Mg: 0.1–0.2; vitamin B1: 0.5%–0.7%; Niacin: 6.1%–7.5%; vitamin B6: 0.4%–2%; vitamin B12: 0.1%–0.6%; and pantothenic acid: 3%). While there is no difference in the prevalence of inadequate intake of vitamin D calculated by CIMI‐Ethiopia and NS, it was in between 15.5% and 17.7% for vitamin A and vitamin C (7.9%–22.9%) among lactating mothers (Table 2).

**TABLE 2:** Frequency and percentage distribution of children and lactating mothers with inadequate energy and nutrient intakes (<RNI and <EAR)

Nutrient	Children (12−23months)								Lactating mother							
	Inadequacy (<RNI) (*N* = 781)				Inadequacy (<EAR) (*N =* 781)				Inadequacy (<RNI) (*N =* 1086)				Inadequacy (<EAR) (*N =* 1086)			
	CIMI		NS		CIMI		NS		CIMI		NS		CIMI		NS	
	*N*	%	*N*	%	*N*	%	*N*	%	*N*	%	*N*	%	*N*	%	*N*	%
Energy (kcal)	781	91.9	708	90.7					968	89.1	922	84.9				
Protein (g)	431	55.2	430	55.1	184	23.6	176	22.9	453	41.7	469	43.1	386	35.6	391	36.0
Iron (mg)	566	72.5	586	75.0	86	11.01	73	9.4	277	25.5	267	24.6	1	0.09	0	0.0
Zinc (mg)	745	95.4	744	95.3	290	37.13	291	37.3	706	65.0	633	58.3	212	19.52	198	18.23
Vitamin A (µg RE)	753	96.4	528	67.6	661	84.6	308	39.4	1056	97.2	863	79.5	1059	97.51	891	82.04
Calcium (mg)	779	99.7	780	99.9	780	99.9	779	99.7	1081	99.5	1085	99.9	1068	98.34	1084	99.82
Magnesium (mg)	126	16.1	134	17.1	138	17.7	150	19.2	47	4.3	44	4.1	45	4.14	44	4.05
Vitamin B1 (mg)	544	69.7	559	71.6	432	55.3	431	55.2	316	29.1	308	28.4	198	18.23	193	17.77
Niacin (mg)	714	91.4	715	91.5	665	85.6	668	85.5	720	66.3	639	58.8	342	31.49	276	25.41
Vitamin B6 (mg)	727	93.1	748	95.8	686	87.8	724	92.7	1033	95.1	1037	95.5	997	91.80	1019	93.83
Vitamin B12 (µg)	778	99.6	774	99.1	746	95.5	741	94.9	1086	100	1085	99.9	1086	100.0	1080	99.44
Pantothenic acid (mg)	735	94.1	698	89.4	‐	‐	‐	‐	1079	99.4	1039	95.7	‐	‐	‐	‐
Vitamin C (mg)	718	91.9	704	90.1	477	61.1	488	62.5	663	61.0	912	83.9	957	88.12	1043	96.04
Vitamin D (µg)	781	100	777	99.5	781	100.	778	99.6	1086	100	1086	100	1086	100.0	1086	100.0

Note

NB: The inadequacy of the 12–23‐month‐old children and lactating women was declared as the energy, protein, and other micronutrients intake was below the recommended nutrient intake (RNI) and estimated average requirement (EAR). For the zinc and iron, the RNI for CIMI was set based on the individual consumption pattern and category of bioavailability; otherwise, for the NS, the low and 5% bioavailability of zinc and iron were used in this study, respectively. The RNI for the 12–23‐month‐old children from the complementary foods was energy (950 kcal (male), 850 kcal (female)), protein (13.5 g), calcium (500 mg), iron (11.6 mg), zinc (8.3 mg), vitamin B1 (0.5 mg), niacin (6 mg), vitamin B6 (0.5 mg), vitamin C (30 mg), pantothenic acid (2 mg), vitamin B12 (0.9 µg), vitamin A (400 µg), vitamin D (5 µg), and magnesium (60 mg). For the lactating mothers, the RNI: energy (2650 kcal), protein (55 g), calcium (1000 mg), iron (30 mg and 15 mg for very low and low bioavailability, respectively), zinc (17 mg low for bioavailability), vitamin B1 (1.5 mg), niacin (17 mg), vitamin C (70 mg), vitamin A (850 µg RE), vitamin B6 (2 mg), pantothenic acid (7 mg), vitamin B12 (2.8 µg), vitamin D (5 µg), and magnesium (270 mg). The EAR for the 12–23‐month‐old children was calcium (500 mg), iron (3 mg), zinc (2.5 mg), vitamin B1 (0.4 mg), niacin (5 mg), vitamin B6 (0.4 mg), vitamin C (13 mg), vitamin B12 (0.7 µg), vitamin A (210 µg), vitamin D (10 µg), and magnesium (65 mg). For the lactating mothers, the EAR: calcium (800 mg), iron (6.5 mg), zinc (10.4 mg), vitamin B1 (1.2 mg), niacin (13 mg), vitamin C (70 mg), vitamin A (900 µg RE), vitamin B6 (1.7 mg), vitamin B12 (2.4 µg), vitamin D (15 µg), and magnesium (265 mg). Inadequacy of the energy and pantothenic acid was not calculated using the EAR, both for the children aged 12–23 months and the lactating women, due to the data inappropriateness and absence of the cut‐point; for protein, 0.87 g kg^‐1^ day^‐1^ and 1.05 g kg^‐1^ day^‐1^ were considered for identifying the EAR cut‐point for declaring inadequacy (FAO/WHO, 2001; FAO/WHO/UNU, 2001a, 2001b; Institute of Medicine (IOM), 2005, 2011).

### 3.3 Sensitivity, specificity, and predictive values of CIMI‐Ethiopia for calculating inadequate energy and other nutrients intake compared to NS

The sensitivity of CIMI‐Ethiopia to identify true inadequate intake of nutrients (i.e., including energy) was between 84.1% and 100%, of which for the energy, protein, and other ten nutrients included in this study, the sensitivity was >95%, for the lactating mothers' group. The least sensitivity to identify true inadequacy in lactating mothers group was observed for magnesium (84.1%). Whereas, the ability of CIMI‐Ethiopia to identify true adequacy (true negative) was from 0% for vitamin B12 to 100% for calcium, for the same study group. However, CIMI‐Ethiopia identified that true negatives (specificity, i.e., true adequate identified) for nutrients (Protein, iron, zinc, vitamin B1, B6, and C) were between 81% and 96%. The positive predictive values of CIMI‐Ethiopia to identify true inadequacy for energy, protein, and 10 nutrients included in this study were above 85%, except that of vitamin A (81.2%), and magnesium and vitamin C (78.7%). However, the negative predictive values were calcium (20%), vitamin C (37.8%), pantothenic acid (71.4%), vitamin B6 (75.5%), and vitamin A (80%); otherwise, it was above 90% for energy, protein, and other nutrients included in this study (Table 3).

**TABLE 3:** Sensitivity, specificity, and predictive values of CIMI‐Ethiopia compared to NS for 12–23‐month‐old children and lactating women below the threshold of inadequate intake (<RNI)

Nutrient	Children (12−23months)				Lactating mothers			
	Sensitivity %	Specificity %	Positive predictive value (%)	Negative predictive value (%)	Sensitivity %	Specificity %	Positive predictive value (%)	Negative predictive value (%)
Energy (kcal)	98.4	71.2	97.1	82.5	99.0	66.5	94.3	92.4
Protein (g)	92.8	90.9	92.6	91.1	88.7	94.0	91.8	91.6
Iron (mg)	93.2	89.7	96.5	81.4	89.1	95.2	85.9	96.4
Zinc (mg)	98.5	67.6	98.4	69.4	98.1	81.2	88.0	96.8
Vitamin A (µg RE)	99.8	10.7	70.0	96.4	99.3	10.8	81.2	80.0
Calcium (mg)	99.9	100.0	100.0	50.0	99.6	100.0	100.0	20.0
Magnesium (mg)	77.6	96.6	82.5	95.4	84.1	99.0	78.7	99.3
Vitamin B1 (mg)	94.3	92.3	96.9	86.5	91.2	95.5	88.9	96.5
Niacin (mg)	98.0	80.3	98.2	79.1	96.9	77.4	86.0	94.5
Vitamin B6 (mg)	99.0	78.8	99.0	47.3	98.7	81.6	99.1	75.5
Vitamin B12 (µg)	100.0	42.9	100.0	42.9	100.0	100.0	100.0	50.0
Pantothenic acid (mg)	98.9	45.8	93.9	82.6	99.8	10.6	96.1	71.4
Vitamin C (mg)	97.4	58.4	95.5	71.4	84.1	99.4	78.7	99.3
Vitamin D (µg)	100.0	0.0	99.5	#DIV/0	100.0	0.0	99.4	‐

Similarly, the sensitivity of CIMI‐Ethiopia to identify true inadequate intake of energy, protein, and other nutrients was >90%, except that of magnesium (77.6%), in the children aged between 12 and 23 months. Whereas, the ability of CIMI‐Ethiopia to identify true negative (Specificity) was from 10.7% for vitamin A to 100% both for calcium and vitamin D. The positive predictive values of CIMI‐Ethiopia to identify true inadequacy for energy, protein, and other nutrients included in this study were above 90%, except for the vitamin A (70%) and magnesium (82.5%), for the same age group. Likewise, the negative predictive values were low for vitamin B12 (42.9%), vitamin B6 (47.3%), calcium (50%), and zinc (69.4%), but it was >70% for energy, protein, and other nutrients included in this study (Table 3).

### 3.4 Average intake, mean differences, and correlation among energy and nutrients intake calculated by CIMI‐Ethiopia and NS

Relatively, results on average energy and nutrient intakes generated using NS and CIMI‐Ethiopia were comparable except that was observed for vitamin A for the children (213.31 µg RE) and mothers (342.23 µg RE) (Tables 4 and 5). There was no difference observed between the results generated using NS and CIMI‐Ethiopia results for vitamin D for the children and vitamin B1 for the lactating mothers' group. According to Lambert and colleagues’ (2018) criterion, the results generated by CIMI‐Ethiopia for energy, protein, iron, zinc, calcium, vitamin B1, niacin, and pantothenic acid produced were from good to very high accuracy categories, for both study groups. The vitamin A results calculated by CIMI‐Ethiopia in terms of NS was moderately accurate regardless of the study groups, whereas low accuracies were observed for the vitamin B6 for the children and vitamin C for the lactating mothers' group (Tables 4 and 5).

**TABLE 4:** Mean (*SD*) and median (25th, 75th) of energy and nutrient intake calculated by CIMI‐Ethiopia and NS, mean differences of calculated nutrient intake by CIMI and NS, categorization of accuracy based on a percentage of mean differences of nutrient intake data (CIMI‐Ethiopia minus NS) in relation to mean NS result, and the Pearson's coefficients of the nutrient intake calculated by CIMI‐Ethiopia and NS (12–23‐month‐old children)

Children (12–23 months) (*n =* 7*81*)								
Nutrient		NS	CIMI	Mean difference between CIMI and NS result	*SD*	Mean difference in % of mean nutrient intake calculated by NS	Accuracy category	*R*
Energy (kcal)	Mean (*SD*) Median (25^th^, 75^th^)	508 (291) 465 (318, 644)	503 (342) 447 (294, 628)	−4.15	186.23	−1.09	Very high accurate	.97*
Protein (g)	Mean (*SD*) Median (25^th^, 75^th^)	14.3 (9.2) 12.7 (8.6, 18.5)	15.0 (11.3) 12.6 (8.6, 18.9)	−0.71	5.68	3.58	Very high accurate	.97*
Iron (mg)	Mean (*SD*) Median (25^th^, 75^th^)	8.9 (5.1) 7.8 (5.4, 11.6)	9.7 (7.4) 8.02 (5.6, 12.0)	0.84	4.36	9.71	Good accurate	.96*
Zinc (mg)	Mean (*SD*) Median (25^th^, 75^th^)	3.54(2.43) 3.06 (2.02, 4.60)	3.53(2.68) 3.05 (1.90, 4.43)	−0.01	1.71	4.19	Very high accurate	.96*
Vitamin A (µg RE)	Mean (*SD*) Median (25^th^, 75^th^)	337 (328) 261 (92.6, 457)	123 (152) 90.3 (30.7, 165)	−213.31	202.21	22.16	Moderate accurate	.96*
Calcium (mg)	Mean (*SD*) Median (25^th^, 75^th^)	82.9 (62.7) 67.8(44.5, 104)	89.7 (73.2) 69.7(45.1, 113)	6.75	43.06	8.94	Good accurate	.96*
Magnesium (mg)	Mean (*SD*) Median (25^th^, 75^th^)	138 (95.1) 120 (80.2, 176)	158 (123) 132 (85.7, 204)	19.66	73.37	16.54	Moderate accurate	.95*
Vitamin B1 (mg)	Mean (*SD*) Median (25^th^, 75^th^)	0.41 (0.27) 0.36 (0.23, 0.53)	0.43 (0.33) 0.37 (0.23, 0.54)	0.02	0.17	6.49	Good accurate	.97*
Niacin(mg)	Mean (*SD*) Median (25^th^, 75^th^)	3.21 (2.17) 2.85 (1.85, 4.11)	3.33 (2.62) 2.85 (1.94, 4.24)	0.12	1.57	5.44	Good accurate	.96*
Vitamin B6 (mg)	Mean (*SD*) Median (25^th^, 75^th^)	0.15 (0.17) 0.11 (0.06, 0.20)	0.19 (0.22) 0.13 (0.07, 0.26)	0.04	0.14	36.98	Low accurate	.92*
Vitamin B12 (µg)	Mean (*SD*) Median (25^th^, 75^th^)	0.11 (0.24) 0.00 (0.00, 0.00)	0.10 (0.21) 0.00 (0.00, 0.00)	−0.01	0.17	‐	‐	.92*
Pantothenic acid (mg)	Mean (*SD*) Median (25^th^, 75^th^)	1.09 (0.74) 0.95 (0.60, 1.42)	0.94 (0.68) 0.81 (0.48, 1.22)	−0.15	0.40	−11.02	Good accurate	.96*
Vitamin C (mg)	Mean (*SD*) Median (25^th^, 75^th^)	12.9 (12.9) 6.8 (3.25, 22.3)	12.1 (12.4) 8.0 (3.27, 17.2)	−0.82	8.03	8.23	Good accurate	.96*
Vitamin D (µg)	Mean (*SD*) Median (25^th^, 75^th^)	0.21 (0.44) 0.00 (0.00, 0.00)	0.22 (0.44) 0.02 (0.01, 0.06)	0.00	0.13	‐	‐	.85*

Note

Nutrients with a very high accuracy (difference expressed as % of NS result: ±0 < 5%), good accuracy (±5%–15%), moderate accuracy (±15%–30%), and low accuracy (±>30%) was adopted (Lambert et al., 2018). For vitamin B12 and vitamin D, the mean difference % was not calculated, as most of these values were zero in NS, which is a denominator for computation.

* All the intake results produced by the CIMI‐Ethiopia and NS are statistically significant at *p*‐value <.001.

**TABLE 5:** Mean (*SD*) and median (25th, 75th) of energy and nutrient intake calculated by CIMI and NS, mean differences of calculated nutrient intake by CIMI and NS, categorization of accuracy based on percentage of mean difference of nutrient intake data (CIMI minus NS) in relation to mean NS result, and the Pearson's coefficients of the nutrient intake calculated by CIMI and NS (Lactating women)

Lactating women (*n *= 1086)								
Nutrient		NS	CIMI	Mean difference between CIMI and NS result	*SD*	Mean difference in % of mean nutrient intake calculated by NS	Accuracy category	*R*
Energy (kcal)	Mean (*SD*) Median (25^th^, 75^th^)	2014 (647) 2040 (1585, 2418)	1943 (622) 1995 (1525, 2356)	−71.04	268.37	−2.49	Very high accurate	.96*
Protein (g)	Mean (*SD*) Median (25^th^, 75^th^)	59.0 (21.6) 58.8 (44.6, 72.1)	59.6 (22.9) 59.7 (43.7, 73.0)	0.60	8.99	1.71	Very high accurate	.96*
Iron (mg)	Mean (*SD*) Median (25^th^, 75^th^)	38.9 (14.8) 38.9 (30.1, 47.0)	39.8 (15.6) 40.4 (29.7, 47.6)	0.85	8.05	3.42	Very high accurate	.95*
Zinc (mg)	Mean (*SD*) Median (25^th^, 75^th^)	15.7 (5.5) 15.9 (12.2, 19.3)	14.8 (5.4) 15.1 (11.4, 17.9)	−0.92	2.91	−4.44	Very high accurate	.96*
Vitamin A (µg RE)	Mean (*SD*) Median (25^th^, 75^th^)	576 (561) 438 (229, 707)	234 (291) 164 (81.7, 297)	−342.23	441.12	−27.09	Moderate accurate	.96*
Calcium (mg)	Mean (*SD*) Median (25^th^, 75^th^)	326 (129) 319 (246, 395)	348 (160) 326 (247, 417)	22.46	83.27	7.08	Good accurate	.94*
Magnesium (mg)	Mean (*SD*) Median (25^th^, 75^th^)	654 (210) 669 (512, 806)	690 (232) 717 (536, 837)	36.02	117.26	6.50	Good accurate	.95*
Vitamin B1 (mg)	Mean (*SD*) Median (25^th^, 75^th^)	1.82 (0.63) 1.84 (1.40, 2.23)	1.82 (0.65) 1.84 (1.37, 2.23)	0.00	0.27	0.96	Very high accurate	.96*
Niacin(mg)	Mean (*SD*) Median (25^th^, 75^th^)	15.9 (5.1) 16.0 (12.8, 18.9)	14.9 (4.93) 15.3 (11.8, 17.9)	−1.05	2.48	−5.74	Good accurate	.95*
Vitamin B6 (mg)	Mean (*SD*) Median (25^th^, 75^th^)	0.78 (0.59) 0.62 (0.39, 0.98)	0.88 (0.61) 0.73 (0.44, 1.15)	0.10	0.31	21.28	Moderate accurate	.94*
Vitamin B12 (µg)	Mean (*SD*) Median (25^th^, 75^th^)	0.05 (0.29) 0.00 (0.00, 0.00)	0.04 (0.19) 0.00 (0.00, 0.00)	−0.01	1.49	‐	‐	.87*
Pantothenic acid (mg)	Mean (*SD*) Median (25^th^, 75^th^)	3.98 (1.64) 3.86 (2.80, 4.90)	3.42 (1.27) 3.42 (2.61, 4.14)	−0.56	0.85	−11.23	Good accurate	.95*
Vitamin C (mg)	Mean (*SD*) Median (25^th^, 75^th^)	47.9 (26.1) 41.5 (31.2, 57.8)	64.9 (31.9) 61.1 (42.8, 82.7)	16.95	20.09	42.06	Low accurate	.88*
Vitamin D (µg)	Mean (*SD*) Median (25^th^, 75^th^)	0.02 (0.16) 0.00 (0.00, 0.00)	0.05 (0.15) 0.03 (0.01, 0.04)	0.03	0.09	‐	‐	.50*

Note

Nutrients with a very high accuracy (difference expressed as % of NS result: ±0 < 5%), good accuracy (±5%–15%), moderate accuracy (±15%–30%), and low accuracy (±>30%) was adopted (Lambert et al., 2018). For vitamin B12 and vitamin D, the mean difference % was not calculated, as most of these values were zero in NS, which is a denominator for computation.

* All the intake results produced by the CIMI‐Ethiopia and NS is statistically significant at *p*‐value <.001.

### 3.5 Correlation of energy and nutrients intake results calculated by CIMI‐Ethiopia and NS

In the Spearman correlation analyses, the highest correlation coefficients (*r*) between CIMI‐Ethiopia and NS were observed for energy, protein, and vitamin B1 (0.97) for children and energy, protein, zinc, vitamin A and B1 (0.96) for lactating mothers group. On the other hand, the lowest *r* was observed in vitamin D (0.85 and 0.5) for the children and lactating mothers, respectively. However, all the correlations were highly significant (*p* <.001) (Tables 4 and 5).

### 3.6 Bland–Altman plots for energy and other nutrients intake calculated by CIMI‐Ethiopia and NS

The Bland–Altman plots constructed for the intakes of energy and most nutrients of the children and lactating mothers showed that the differences in the results produced by CIMI‐Ethiopia and NS approached zero, and most of these differences were within the acceptable limits (± 1.96SD) (Y‐axis). Despite the average mean differences for the vitamin D and vitamin B12 approaching to zero (y‐axis) in both children and mothers, their Bland–Altman plots showed that the individual values of the mean differences were scattered in irregular patterns. However, the differences in the Bland–Altman plots constructed for vitamin A were dispersed and their average mean difference was far from zero. The higher the vitamin A intake in NS, the greater the underestimation of the calculated value by CIMI‐Ethiopia (Appendix S2, Figures S2 and S3).

## 4 DISCUSSION

The present study focused on assessing the validity of CIMI‐Ethiopia software, a calculator for inadequate micronutrient intake, which is constructed based on 93 commonly consumed food items by the Ethiopian population, using 24‐h dietary recall data of 12–‐23‐month‐old children and lactating mothers collected from rural Genta Afeshum district, Tigray, northern Ethiopia.

Most of the average nutrient and energy intake calculated by CIMI‐Ethiopia and NS were comparable, except for vitamin A, both for children and lactating mothers, and vitamin C in the lactating mothers. These slight variations of the vitamin A intake calculated by CIMI‐Ethiopia and NS are in line with the results observed in the concurrent validation study conducted in Ethiopia, and CIMI validation studies conducted in Indonesia and Tanzania (Bosha, Lambert, et al., 2019; Jati et al., 2014; Lambert et al., 2018). The reason for the variation in the vitamin A calculated by the two software in the present study is mainly that the maximum selectable amount in the “butter and oils” group of the CIMI‐Ethiopia is 30 g unlike in the NS, in which an unlimited amount can be entered (data not shown). For instance, excluding the data of the lactating mothers (*n* = 81) who consumed >30 g from oil and related group in both the methods, then the correlation coefficient increased by 0.12. Thus, increasing the maximum amount set in CIMI‐Ethiopia that can be selected in the specific food group can improve the accuracy to calculate vitamin A precisely. Whereas, the reason for the higher vitamin C intake calculated by CIMI‐Ethiopia compared to the NS for the lactating mothers was due to the great share (43.8%) of tomatoes in the ‘other vegetables’ group, but which were less consumed concerning the cabbage in the study area, which has a relatively lower vitamin C. This leads to a higher vitamin C content for the ‘other vegetables’ food group in the CIMI‐Ethiopia. Therefore, deselection of not consumed foods items within the specific group of CIMI‐Ethiopia will probably overcome the problem and should be implemented in the future CIMI‐Ethiopia use.

However, the protein, iron, calcium, magnesium, vitamin B1, and B6 intakes calculated by CIMI‐Ethiopia were slightly higher than that of NS. The plausible reason for the higher protein, iron, and calcium values estimated by CIMI‐Ethiopia is perhaps due to the consumption of a high amount of peas, although peas' contribution to the relevant food group is only 12.4%, which does not represent the real share of peas in this group. Peas have got a lower content of these nutrients compared to most food items in the group. Besides this, the intervals to set up the amount with the slider get larger if the consumption is increasing in most of the food groups in CIMI‐Ethiopia. Therefore, the selection of the slider either below or above the estimated amount of the food group will depend on the nearest amount set up in the sliders. So if the amount approaches the upper slider, then automatically the upper slider will be selected and this will lead to an overestimation of the intake of specific nutrients when compared to NS. The latter could also work for magnesium and vitamin B1 in pepper and egg food groups, respectively. Ethiopian kale, which has got a higher vitamin B6 content than other vegetables in the ‘green leaves’ food group, contributed 58.4% to this group. But Ethiopian kale was less consumed in the studied area, and this leads to an overestimation of vitamin B6 intake in CIMI‐Ethiopia.

The prevalence of inadequate intakes (<RNI) of energy and most nutrients among the children and lactating mothers identified by CIMI‐Ethiopia and NS was in the same range, except the vitamin A for the children and lactating mothers and vitamin C only for the lactating mothers' group. Likewise, except the vitamin A, the number (%) of participants below the EAR calculated by CIMI‐Ethiopia and NS were also comparable in both study groups. The reasons mentioned for divergent vitamin A and C results are also applied here. In addition, averaging the values for vitamin A of the food items in the ‘butter and oils’ group leads to a reduced intake calculation by CIMI‐Ethiopia.

According to Lambert and colleagues (2018), the vitamin A intake calculated by CIMI‐Ethiopia for the children and lactating mothers groups had moderate accuracy (Lambert et al., 2018). The results found for vitamin A in the validation study conducted in southern Ethiopia and Tanzania were also similar (Bosha, Lambert, et al., 2019; Lambert et al., 2018). Likewise, CIMI‐Ethiopia calculated vitamin B6 intake with low accuracy for the children and moderate accuracy for lactating mothers group. This is because the intake of vitamin B6, calculated by the two software, in both study groups was very small, but the average with small differences (0.04 and 0.1) between them was magnified when these differences were computed in terms of % NS (36.9% and 21.3%). The moderate accuracy of the magnesium intake calculated for the children by CIMI‐Ethiopia might be related to the average of the ‘beans’ group which was higher than the commonly consumed pea entered individually in NS. Whereas, the low accuracy in the vitamin C calculated for the lactating mothers could be due to the relatively high vitamin C content of the food group ‘other vegetables’ in CIMI‐Ethiopia. Twenty‐four‐hour recall data of the study participants showed that food items of this food group were consumed in different proportions compared to the standard composition of food items in this group. Thereby, CIMI‐Ethiopia vitamin C results are higher than the calculation by NS, which is based on single food items.

Strong positive correlations between CIMI‐Ethiopia and NS were found in all the nutrients and energy for both study groups. The positive correlation coefficients between CIMI‐Ethiopia and NS in protein, iron, zinc, and vitamin A for the children and energy, protein, iron, and zinc for the lactating mothers in the present study were higher than those earlier reported for the children and women in the respective nutrients in the Indonesian CIMI validation study (Jati et al., 2014). This could be attributed to the smaller sample size in the Indonesian study. Furthermore, the correlation coefficients of all nutrients in the present study were higher than those reported in the validation study for CIMI in Tanzanian women (Lambert et al., 2018). Whereas, a CIMI validation study conducted in Ghana reported a higher positive correlation between the CIMI and NS results produced for energy, protein, iron, zinc, and vitamin A intake for the children, women, and men groups. These variations could be observed due to differences in the total number of food items used to create the food groups, and also the food groups created for the specific CIMI version (Jati et al., 2014; Wald et al., 2017). A strong positive correlation was also observed in energy, protein, and micronutrient (*r* = .90–.97) results produced by CIMI‐Ethiopia and NS in southern Ethiopia, except for the slight inconsistencies observed with the results found in the present study (Bosha, Lambert, et al., 2019). This is probably due to the differences in the staple crops majorly consumed by the communities, which are the root and tuber crops in the southern and the cereals in the northern parts of Ethiopia. In addition to this, the amount of dietary energy and the entire nutrients intake by the lactating mothers were higher in the present study compared to the mothers participating in the validation study in south Ethiopia. This indicates that the amount of food consumed from different food items is higher in our case, which leads to sliding to the nearest defined values in CIMI‐Ethiopia, unlike the entry in the NS, which allows the entry of the actual amount consumed in the corresponding food items individually. Thus, reducing the interval between the sliders in the right margin, besides implementing of the selection/deselection of food items within the food groups, will further improve the results produced from CIMI‐Ethiopia. Improving these features in CIMI‐Ethiopia will further increase the ability to precisely calculate the total energy, protein, and other micronutrients and their inadequacy from the diet consumed by the individuals.

Unlike the energy and other nutrients, the Bland–Altman plots constructed for the vitamin D and vitamin B12 intakes showed that the individual mean differences are irregularly scattered, despite the average mean differences approaching zero for both the children and lactating mothers. This could happen because the intake values for vitamin D and vitamin B12 were almost nil because the consumption of animal source foods, which are the main source of vitamin D and vitamin B12, was insignificant. Therefore, the small differences in the intake calculated between CIMI‐Ethiopia from NS are magnified in general. Similar results were observed in vitamin D in the CIMI‐Ethiopia validation in southern Ethiopia (Bosha, Lambert, et al., 2019). Otherwise, the ability of CIMI‐Ethiopia to calculate the inadequate intake of these nutrients is comparable with that of the reference, NS. More importantly, the mean differences for the vitamin A result between the CIMI‐Ethiopia and NS were dispersed, and the average of these differences was far from zero in the Bland–Altman plots (Appendix S2, Figures S2 and S3). This could be because the maximum amount in the ‘butter and oils’ group is limited to 30 g, whereas NS allows the input of values without any restriction. The exclusion of study participants, who consumed more food items than 30 g of the butter and oils group, resulted in an increased relationship between the results produced by NS and CIMI‐Ethiopia.

Our study generally illustrates that CIMI‐Ethiopia validly calculates the energy and most nutrient intake. It also identifies the inadequacy of micronutrient intake of individuals using the cutoff points of RNI of FAO/WHO. Furthermore, the results of energy and nutrient intake calculated by CIMI‐Ethiopia can be used to identify the inadequacy of the individual using the cutoff point of the EAR. Unlike other dietary assessment techniques, CIMI‐Ethiopia provides results immediately after an interview is completed, which enables the enumerator to provide direct feedback to the interviewee and suggest dietary behavior improvements considering the socioeconomic status of the household, the culture of the society, and availability of potential and alternative food items in local farms and/or market. A recent study on assessing the acceptability and speed of application of CIMI‐Ethiopia in a rural setting revealed that the time used to collect 24‐h dietary recall data using CIMI‐Ethiopia was shorter than the conventional paper‐based 24‐h recall data collection method. Moreover, more than two‐thirds of the study participants and the data collectors preferred CIMI‐Ethiopia to the conventional method (Bosha, Desalegn, et al., 2019).

Diagnostic instruments are usually evaluated by their sensitivity and specificity (Parkih et al., 2008). Sensitivity is the ability of a test to correctly classify an individual intake as ‘inadequate’ for a specific nutrient. Accordingly, the sensitivity of CIMI‐Ethiopia to identify true inadequate intake of energy, protein, and other nutrients included in this study, except for magnesium and vitamin C (84.1%) in the lactating mothers, was>85%.

Similarly, the sensitivity of CIMI‐Ethiopia to identify true inadequate intake of energy, protein, and other nutrients was >90%, except that of magnesium (77.6%), in the children aged between 12 and 23 months. These indicated that CIMI‐Ethiopia is highly sensitive to identifying inadequate intake of energy, protein, and most nutrients included in this study. In support of this evidence, a validation study on a rapid test kit for identifying SARS‐CoV‐2 used a cut‐off of 80% sensitivity to judge the tool as highly sensitive (Fernandez‐Montero et al., 2021).

Furthermore, the positive predictive values of CIMI‐Ethiopia to identify true inadequacy for energy and all nutrients were above 90% for 12–23‐month‐old children, except for vitamin A (70%) and magnesium (82.5%). Moreover, the positive predictive values of CIMI‐Ethiopia to identify true inadequacy for vitamin C and magnesium were 78.7% and 81.2% for vitamin A; otherwise, for energy, protein, and other nutrients, it was >85%. These showed that CIMI‐Ethiopia identified true inadequacy highly with less missing of true inadequacy for energy, protein, with a moderate level of vitamin A, and magnesium, in both study groups. Considering the results found on the sensitivity and positive predictive values for a range of nutrients coupled with the purpose of CIMI‐Ethiopia, which is identifying the inadequacy of micronutrients, CIMI‐Ethiopia can be used as a screening tool to identify inadequate intake, both for the lactating mothers and 12–23‐month‐old children in Tigray region and similar settings in Ethiopia.

Whereas, specificity is an indicator to decide whether a given diagnostic kit/machine can be used as a confirmatory test to identify real inadequate intake of energy, protein, and other nutrients of interest by CIMI‐Ethiopia. Accordingly, the ability of CIMI‐Ethiopia to identify true adequate intake was >80%, for protein, calcium, iron, magnesium, vitamin B1, and niacin, for the 12–23‐month‐old children, and also, for protein, iron, calcium, magnesium, vitamin B1, B6, B12, and C, for the lactating mothers' group. Fernandez‐Montero et al. (2021) also mentioned that the cutoff for high specificity of a COVID diagnostic tool was 97% and above. Accordingly, the ability of CIMI‐Ethiopia to identify the true adequacy of calcium and magnesium in both study groups, vitamin B12 and vitamin C additionally for the lactating mothers' group, was high. Furthermore, the ability of CIMI‐Ethiopia to identify the adequacy of protein, vitamin B1, and niacin for the 12–23‐month‐old children and protein, iron, zinc, vitamin B1, and B6 for the lactating mothers' group was moderate.

In addition to the specificity results of CIMI‐Ethiopia, the negative predictive values for energy, protein, iron, vitamin A, magnesium, vitamin B1, and pantothenic acid; and energy, protein, iron, zinc, magnesium, vitamin B1, C, and niacin were >80%, for the 12–23 months old children and lactating mothers group, respectively. On the contrary, our findings showed that CIMI‐Ethiopia was poor to identify adequate intake of vitamin A and D, in both the study groups. The reason for vitamin A mentioned earlier also works here. However, for vitamin D, the proportion for the adequate intake was very small (Table 2). The specificity and negative predictive results found for CIMI‐Ethiopia to identify true nutrient adequacy were moderate or high, regardless of the study group. Therefore, CIMI‐Ethiopia can be used as a confirmatory assessment tool for a range of nutrients despite the purpose of developing CIMI application and adapting to the Ethiopian context was to screen inadequacy. Yet, further efforts to enhance the current ability of CIMI‐Ethiopia to accurately calculate nutrient intake (including energy) can improve its usability as a screening and confirmatory tool to identify inadequate intake for a range of nutrients.

On top of these, most dietary assessment and intervention studies have been using diet diversity scores to assess the dietary quality at the household and individual levels (FAO & FHI^360^, ^2016^). However, this technique only focuses on the assessment of the diversity of food groups consumed regardless of the amount of intake from the food groups, which could be a challenge to predict the adequacy of micronutrient intake from individual diets. To overcome this problem, many studies have used a cutoff of ≥15 g to count the food group as consumed in women of reproductive age (FAO & FHI^360^, ^2016^). But, still, the controversy remains on how quantification is practical in real qualitative diet diversity assessment techniques unless the diet diversity data is extracted from other quantitative dietary intake data. Thus, CIMI‐Ethiopia can be used to generate the qualitative diet diversity, by producing much more results on the diet quality of an individual and overcoming the challenges encountered by DDS. CIMI‐Ethiopia can also be used in cross‐sectional and/or longitudinal/intervention studies, in the situation where the conventional diet diversity score is not appropriate or requires the quantitative dietary assessment methods. These indicate that CIMI‐Ethiopia developed for the Ethiopian population is applicable for dietary micronutrient assessment on an individual level in a rural setting of the region. This needs further piloting of CIMI‐Ethiopia in the Health Extension system at the rural Health posts before implementing it at a specific region and country‐level at large. The accumulation of this individual data can provide information on the dietary nutrient intake in special population groups for designing targeted micronutrient prevention and intervention activities. In general, CIMI‐Ethiopia can be an important tool for nutrition and related researchers, practitioners, policymakers, and planners in the country.

Our present study has much strength. Among these is the first of its kind in Ethiopia, to develop the dietary assessment software (CIMI‐Ethiopia), which is constructed using commonly consumed foods in rural Ethiopia. The validity of the results produced by CIMI‐Ethiopia was assessed using quantitative dietary data collected from the vulnerable groups for malnutrition (young children and lactating mothers) with a large sample size in rural Ethiopia. The data collection periods, which are lent fasting and nonfasting periods, were also considered to include more diversified food items and to increase the trustworthiness of this validation study.

However, the following are the limitations of this study. CIMI‐Ethiopia didn't account the contribution of breast milk during the calculation of energy and nutrient intake for breastfed children. The limitation to the 24‐h dietary recall technique could be also committed in this study, as it is not a gold standard dietary data collection method. Furthermore, the study was conducted only in a rural area and one administrative region in Ethiopia, out of the 11 regions.

## 5 CONCLUSIONS

In conclusion, CIMI‐Ethiopia adapted for the Ethiopian population estimates the average nutrient intake and identifies precisely the inadequate micronutrient intake on an individual level using FAO/WHO RNI directly after the interview. This can help data collectors using CIMI‐Ethiopia to provide immediate nutrition advice and suggest context‐based dietary improvements. Furthermore, EAR and other dietary reference methods can also be used to identify the level of inadequacy from individual dietary data or to provide information on dietary nutrient intake at the population level from the accumulation of individual data for designing targeted micronutrient prevention and intervention activities.

CIMI‐Ethiopia can also be used in cross‐sectional and/or longitudinal/intervention studies, focused on quantitative dietary assessment. Further improvement in the accuracy of CIMI‐Ethiopia should include functioning of “deselection”, the option for not consumed food items within the 31 food groups and enlargement of the maximum selectable amount that is considered to be consumed in the ‘butter and oils’ group. Before full implementation of CIMI‐Ethiopia in the specific region and country at large, piloting in the health extension system of rural health posts should be done, for further improvement and usage in rural settings.

## CONFLICT OF INTEREST

The authors declare that they have no competing interests.

## ETHICAL APPROVAL

This study was conducted according to the guidelines laid down in the Declaration of Helsinki, and all procedures involving human subjects were approved by the Ethics Review Board of Hawassa University, Ethiopia (reference number IRB/078/09) and the Ethical Review Committee of Landesärztekammer Baden‐Württemberg, Germany (dated on February 9, 2017). Informed oral consent was secured from the individual mother for the involvement of herself and her paired child involved in the study.

## Supporting information

App S1

App S2

## Data Availability

The data that support the findings of this study are available from the corresponding author upon reasonable request.
