# Supplementary material for: Calculator for inadequate micronutrient intake for Ethiopia (CIMI‐Ethiopia): Validation of the software for lactating mothers and their children under 2 years
Source: Food Sci Nutr. 2022 Jun 3;10(10):3323–37. doi: 10.1002/fsn3.2922 (PMC9548364; doi:10.1002/fsn3.2922)
Supplement: Supplementary file 1 — App S1 [file FSN3-10-3323-s001.docx]

$$\frac{Individual nutrient content \left( g \right)of specific food item out of 100g from EFCT database=(Individual nutrient content \left( g \right)of specific food item out of 100g from USDA database*Dry matter of individual food item content \left( g \right) out of the 100g from EFCT database)}{Dry matter of individual food item content \left( g \right) out of the 100g from USDA database}$$

Figure S1 Calculation of concentration for nutrients missing by adjusting with the moisture contents of the food items

$Z=\frac{\sum_{i=1}^{n} [X_{i}-Y_{i}]}{n}$[ Equation a] $R=[\frac{Z}{\frac{\sum_{i=1}^{n} Y_{i}}{n}}]*100$ [Equation b]

where Z is the average mean difference of each nutrient and energy, X is the nutrient value of each participant calculated by CIMI-Ethiopia, Y is the nutrient value of each participant calculated by NS, n is the number of participants, R is the average mean difference % of each nutrient and energy. Based on this, the mean difference % expressed in terms of NS was used to categorize the nutrient intake result produced by CIMI-Ethiopia as very high accurate (+/-0<5%), good accuracy (+/-5-15%) moderate accuracy (+/-15-30%) and low accuracy (+/->30%) [12].

Sensitivity = [a/(a+c)]×100 [Equation C]

Specificity = [d/(b+d)]×100 [Equation D]

Positive predictive value (PPV) = [a/(a+b)]×100 [Equation E]

Negative predictive value (NPV)=[d/(c+d)]×100 [Equation F]

Where, a- true positive, b- false positive, c- false negative, and d- true negative

Source: Trevethan, 2017
