# Supplementary material for: Calculator for inadequate micronutrient intake for Ethiopia (CIMI‐Ethiopia): Validation of the software for lactating mothers and their children under 2 years
Source: Food Sci Nutr. 2022 Jun 3;10(10):3323–37. doi: 10.1002/fsn3.2922 (PMC9548364; doi:10.1002/fsn3.2922)
Supplement: Supplementary file 2 — App S2 [file FSN3-10-3323-s002.docx]

*
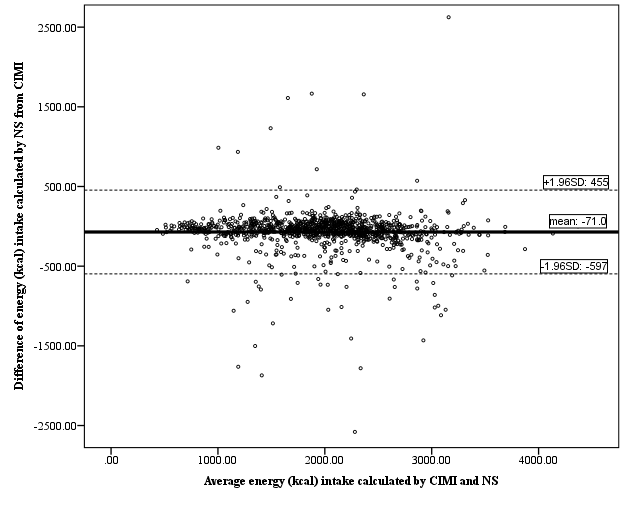

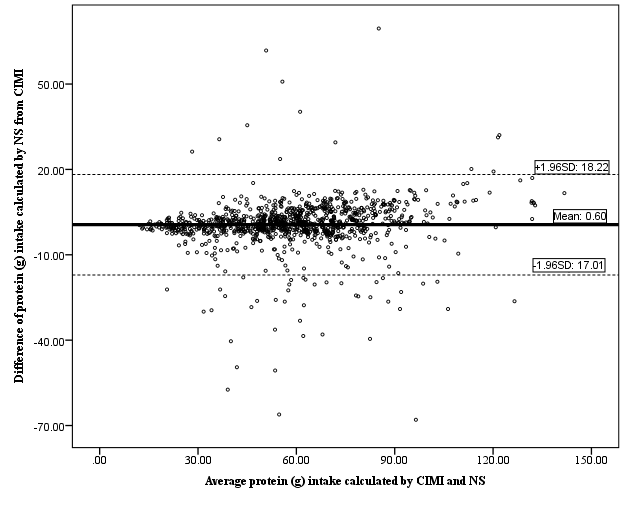

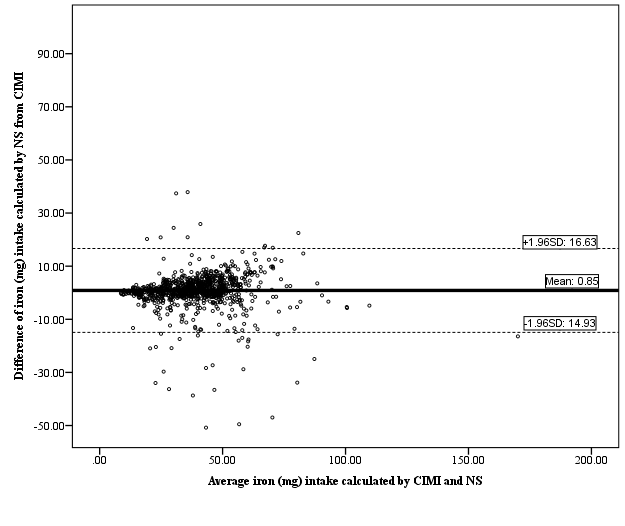

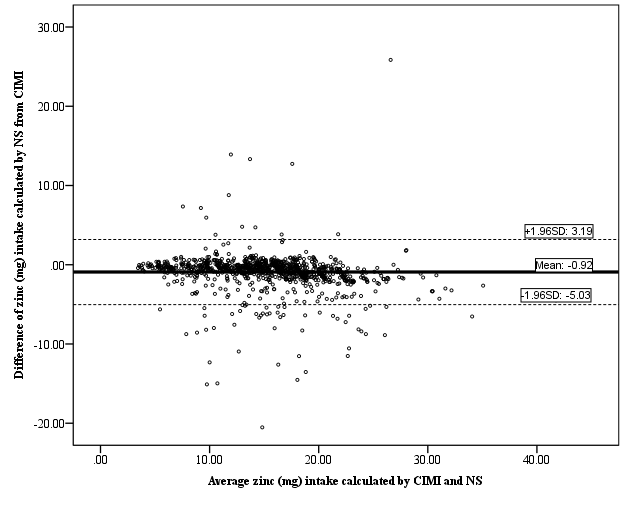

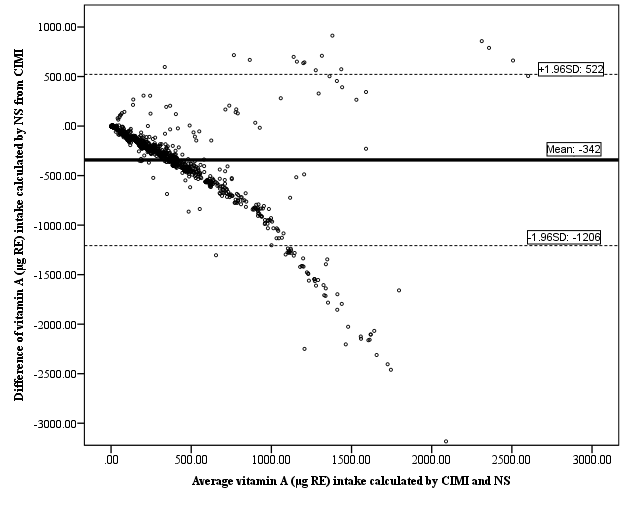
****
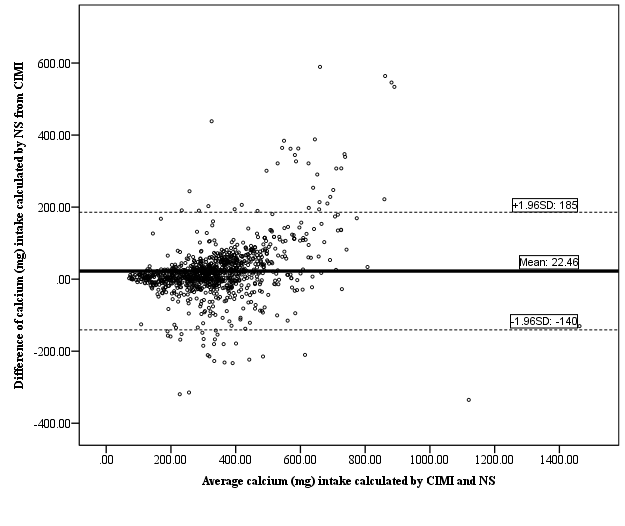

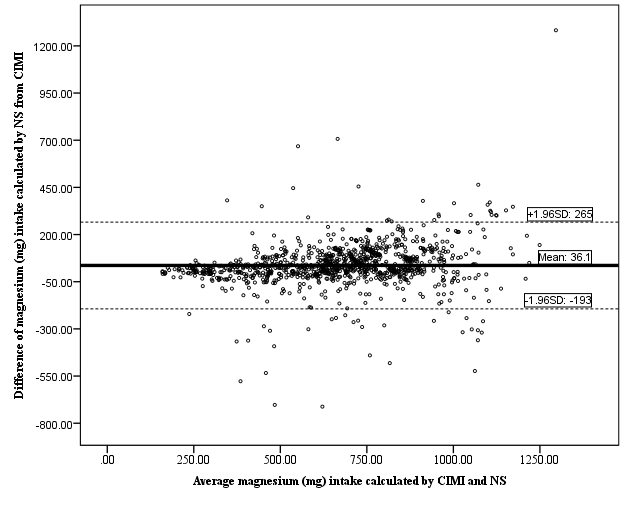

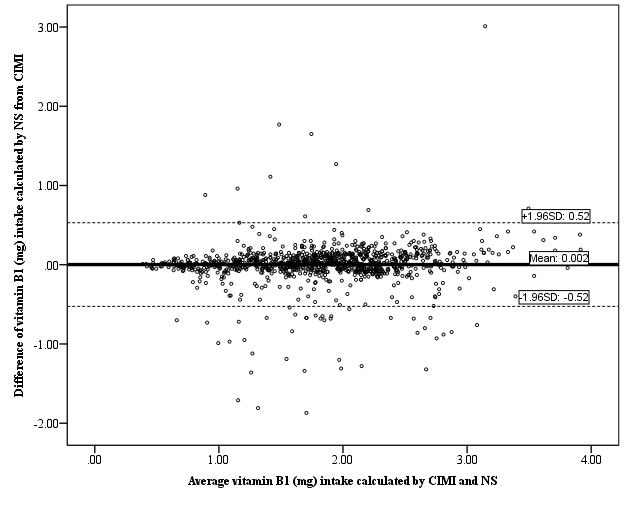

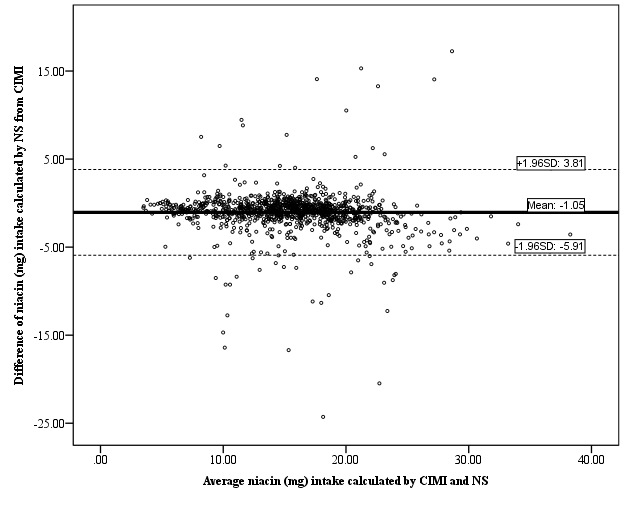

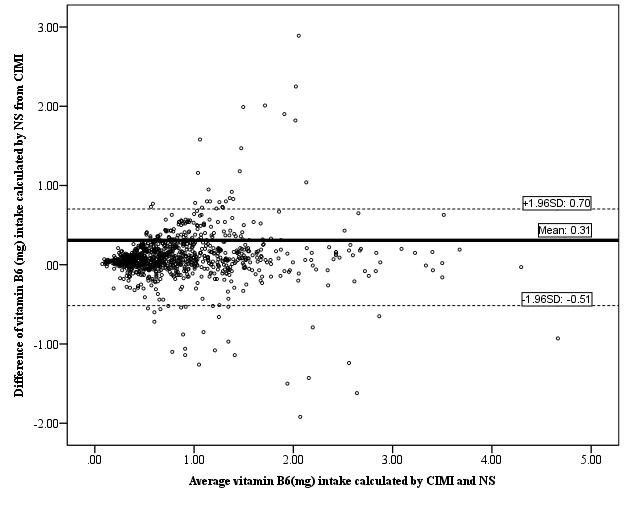

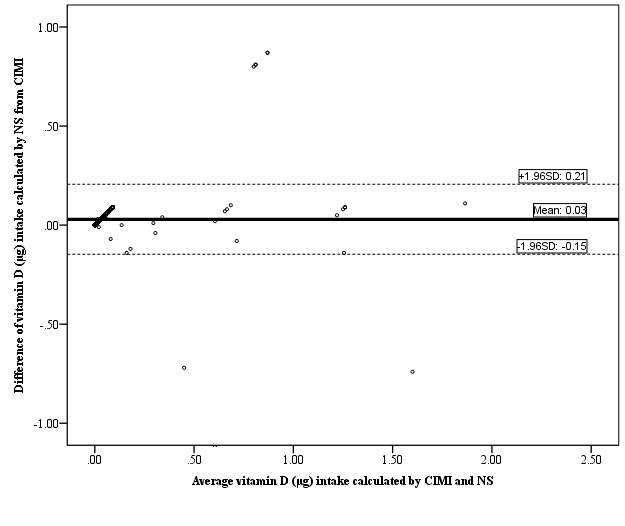

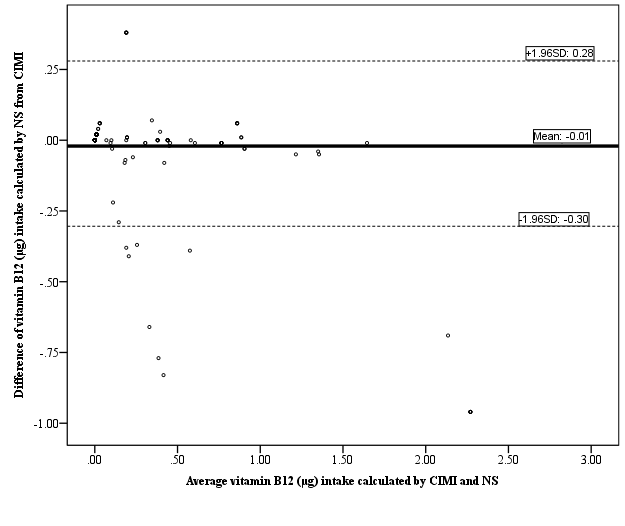

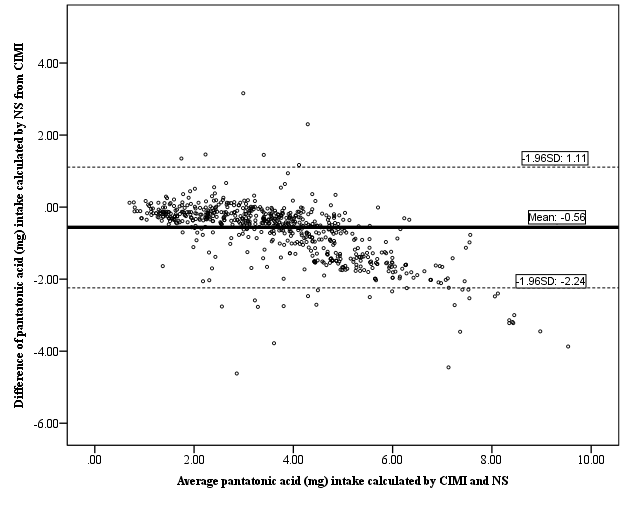

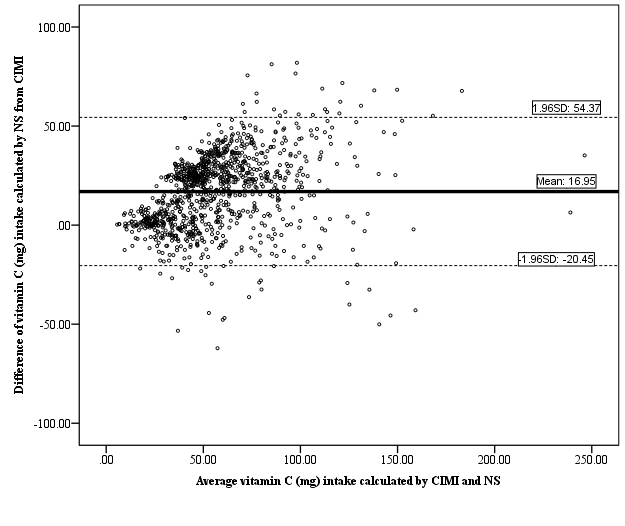
***

Figure S2: Bland-Altman-Plots for energy, protein and micronutrients for the lactating women group (n= 1086). The solid dark horizontal line in these scatter plots is the average mean difference of the intake calculated by NS from CIMI Ethiopia, which the mean bias, and the solid light horizonatal lines are the the limit of agreement (+ 1.96 SD). The values in the y-axis are the mean differences, whereas the values in the x-axis are the average of the values calcuated byCIMI Ethiopia and NS.

*
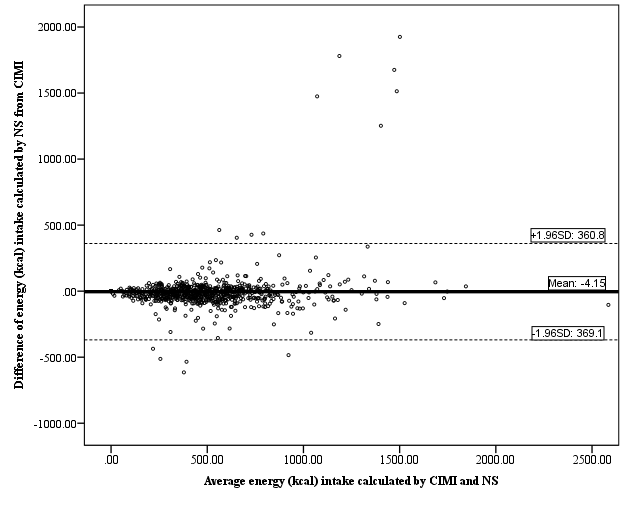

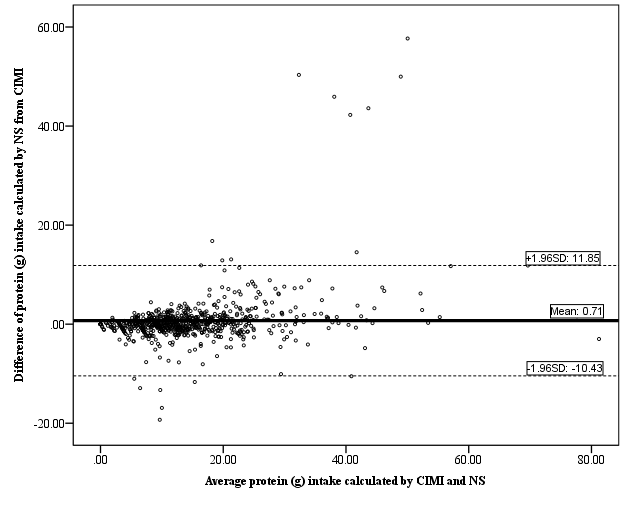

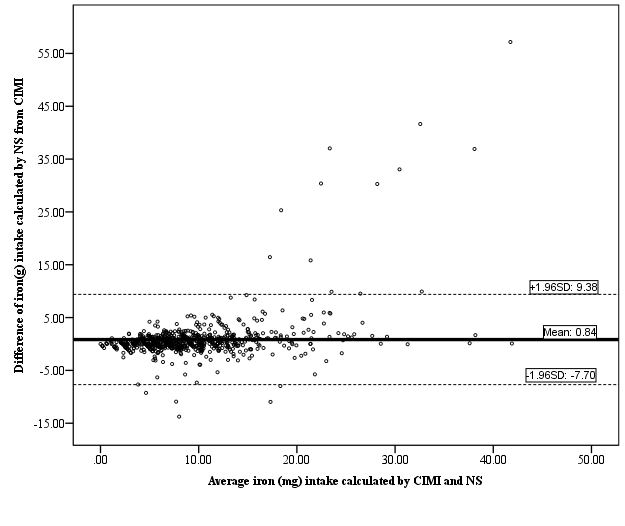

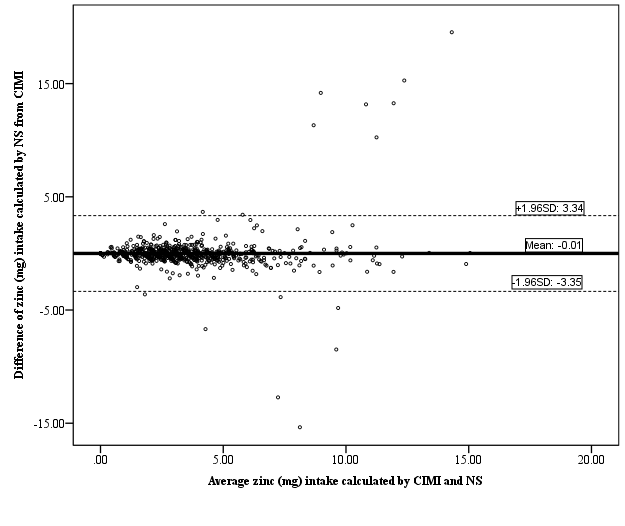

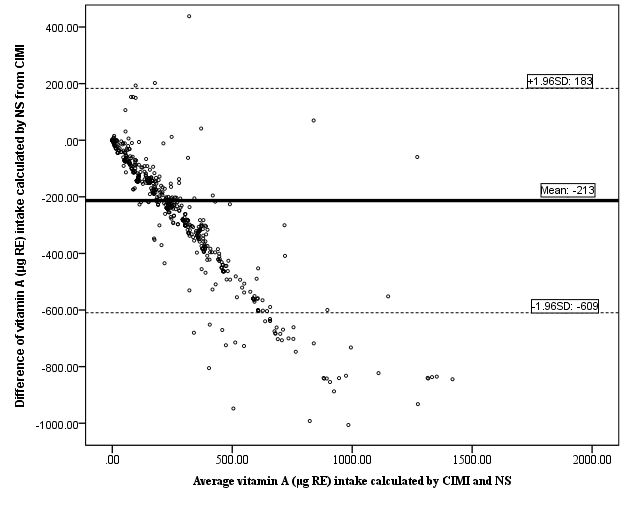

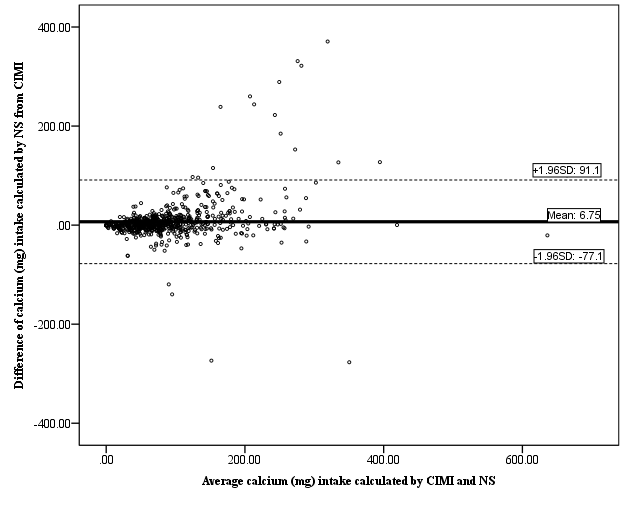

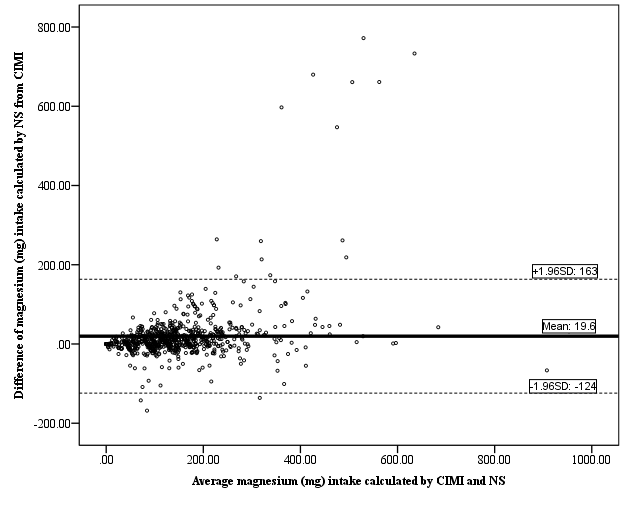

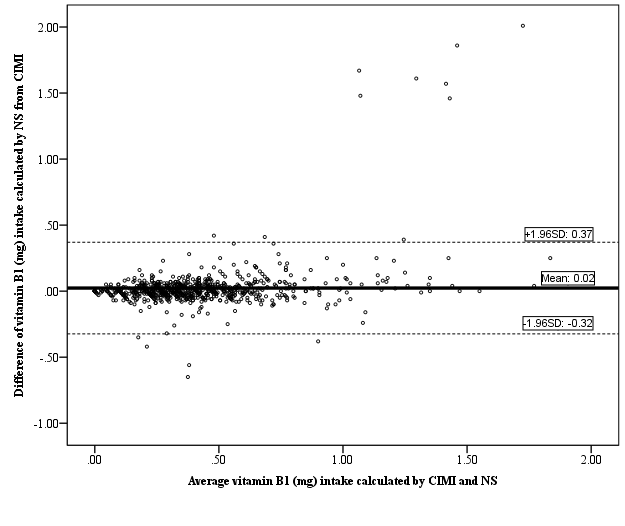

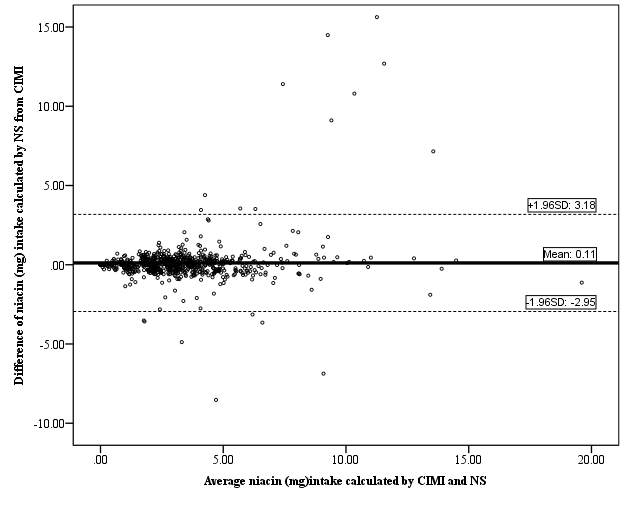

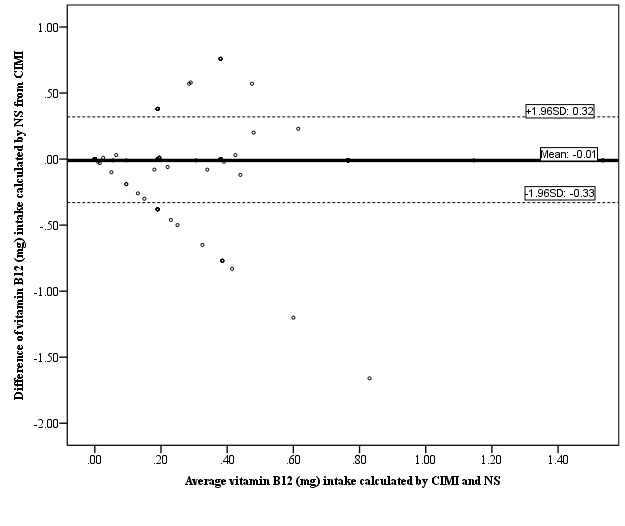

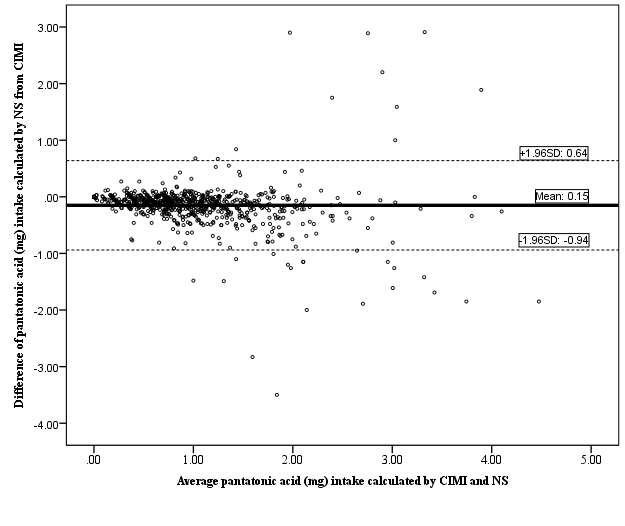

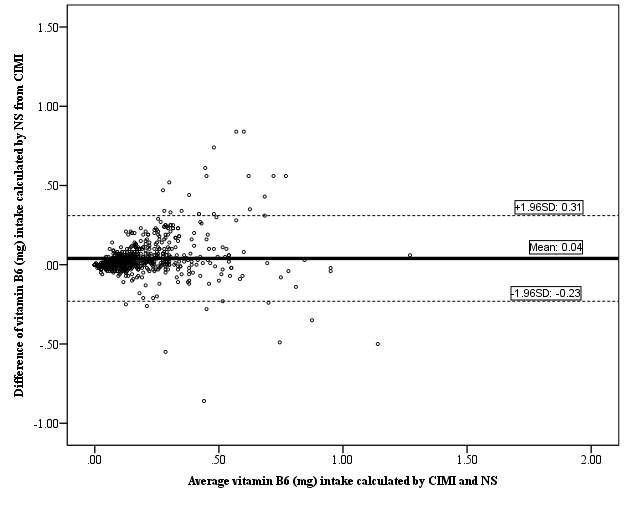

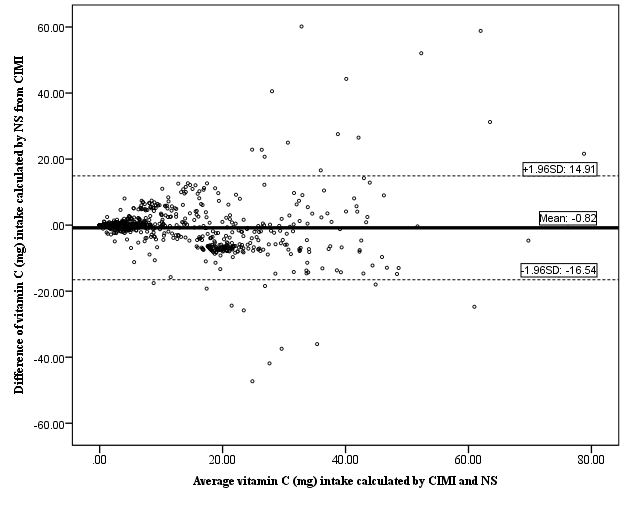

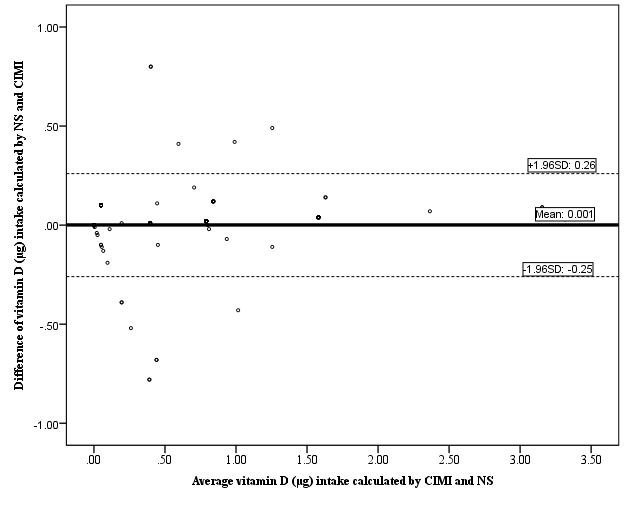
*

Figure S3: Bland-Altman-Plots for energy, protein and micronutrients for the young children aged 12-23 months old (n= 781). The solid dark horizontal line in these scatter plots is the average mean difference of the intake calculated by NS from CIMI Ethiopia, which the mean bias, and the solid light horizonatal lines are the the limit of agreement (+ 1.96 SD). The values in the y-axis are the mean differences, whereas the values in the x-axis are the average of the values calcuated byCIMI Ethiopia and NS.

$Z=\frac{\sum_{i=1}^{n} [X_{i}-Y_{i}]}{n}$[ Equation a] $R=[\frac{Z}{\frac{\sum_{i=1}^{n} Y_{i}}{n}}]*100$ [Equation b]

where Z is the average mean difference of each nutrient and energy, X is the nutrient value of each participant calculated by CIMI-Ethiopia, Y is the nutrient value of each participant calculated by NS, n is the number of participants, R is the average mean difference % of each nutrient and energy.

Based on this, the mean difference % expressed in terms of NS was used to categorize the nutrient intake result produced by CIMI-Ethiopia as very high accurate (+/-0<5%), good accuracy (+/-5-15%) moderate accuracy (+/-15-30%) and low accuracy (+/->30%) [12].
